# Analysis of the Correlation Between Postoperative MRI Findings, Patient-Reported Outcome Measures, and Residual Pain After Arthroscopic TFCC Repair—A Pilot Study

**DOI:** 10.3390/jcm14113729

**Published:** 2025-05-26

**Authors:** Francesca von Matthey, Franziska Hampel, Georg Feuerriegel, Klaus Woertler, Alexandra Gersing, Helen Abel

**Affiliations:** 1 Department of Trauma Surgery, Klinikum Rechts der Isar, Technische Universität München, 81675 Munich, Germany; 2 Musculoskeletal Radiology Section, TUM School of Medicine, Technical University of Munich, 81675 Munich, Germany

**Keywords:** TFCC (triangular fibrocartilage complex), arthroscopy, MRI (magnet resonance imaging), wrist pain, PROM (patient-reported outcome measurement)

## Abstract

**Background:** Triangular fibrocartilage complex (TFCC) tears are a common source of ulnar-sided wrist pain. Surgery has to be performed in case of instability, pain, or if non-operative treatment fails. Overall, the results are very good. However, some patients still suffer from pain after surgery. Post-operative MR imaging can reveal potential pathologies but it needs to be assessed whether depicted changes are normal or whether these findings have a clinical significance. Therefore, the purpose of this study was to evaluate postoperative MR imaging and the function of the patients’ wrists in order to assess which postoperative changes are correlated with pain. **Patients and Methods:** All patients with a TFCC lesion who were treated arthroscopically at our hospital between January 2012 and December 2016 were retrospectively enrolled. Seventeen patients with complete data sets were enrolled. Post-operative MRI examinations needed to be performed within 24 months after arthroscopy. The mean magnet resonance imaging (MRI) follow-up was 22 months. The average clinical follow-up was 27.3 months. Age, gender, pain level, PROM scores (Munich Wrist Questionnaire, MWQ), follow-up interval, and TFCC classification (Palmer) were documented. The patients underwent a clinical examination and MR imaging. **Results:** Ten patients (59%) had scar tissue at the triangular fibrocartilaginous complex (TFCC) and nine (53%) had an effusion in the ulnar recess. These findings were not necessarily associated with pain, as six patients without pain and four with pain had scar tissue at the TFCC and six patients without pain and three with pain showed an effusion in the ulnar recessus. Bone marrow edema could be found in the lunate of five patients (29%) (three with pain, two without pain) and in the distal radial ulnar joint (DRUJ) of one patient (6%) with pain. However, typical degenerative changes were not necessarily associated with pain. **Conclusions:** This present study is the first study correlating postoperative MRI findings after arthroscopic assisted TFCC surgery with both pain and function. Bone edema seems to be associated with pain, whereas scarring at the TFCC is visible on MRI but is not necessarily associated with pain.

## 1. Introduction

Triangular fibrocartilage complex (TFCC) tears are a common source of ulnar-sided wrist pain. Patients suffer from reduced grip strength, pain, and impaired wrist function. Moreover, the TFCC contributes substantially with its foveal attachment to the stability of the DRUJ [1,2].

Non-operative treatment of TFCC lesions can be tried if the symptoms include primarily pain but not a DRUJ instability. The non-operative treatment consists of rest, splinting, physiotherapy, modification of the activities, and general antiphlogistica [3]. However, if nonoperative methods fail, surgery has to be considered. An arthroscopic, arthroscopic-assisted, or open repair can be performed. Although the results of the current literature reflect a good overall postoperative outcome after arthroscopic TFCC repair, as can be seen in refs. [1,2,4,5,6,7,8,9], the existing studies are very inhomogeneous; for example, different surgical procedures are compared and follow-up times vary (from 11.1 months to 20 years [2,6,7,8]). This is most likely due to the comparatively small number of patients and the demanding surgical therapy.

However, after arthroscopy and TFCC surgery, a small percentage of patients still suffer from pain or declare that the clinical findings remain unchanged after surgery [4,5,9]. These patients still have pain and limited range of motion (ROM) after arthroscopic surgery and TFCC repair or resection. In this case, for example, a re-rupture of the TFCC should be considered, but other differential diagnoses must also be excluded. MRI imaging is often used as the imaging procedure in this case. The problem here, however, is that the MRI scans vary in image quality after surgery. Analyzing these MRI scans, several problems appear regularly. One of the main problems is that a distinction must be made between normal postoperative changes and newly occurring pathologies with clinical significance. This is important in order to reveal morphological features that would indicate the need for a surgical revision. The purpose of this study was to evaluate postoperative MRI scans as well as the function of the patients’ wrists in order to assess which postoperative pathologies are correlated with pain or loss of function.

## 2. Materials and Methods

### 2.1. Study Population and Data Collection

This retrospective cohort study was approved by the local ethics committee (Ethics Commission of the Medical Faculty, Technical University of Munich, Germany; Ethics proposal number 327/15s), and all patients gave their written informed consent prior to participation.

After a written declaration of consent, all patients with a TFCC lesion and who were treated arthroscopically at our hospital between January 2012 and December 2016 were consecutively enrolled. Exclusion criteria were incomplete documentation, age under 16 years, general contraindications for MR imaging (e.g., pacemaker or other metal implants), incomplete image acquisition (e.g., due to motion artifacts or premature termination of the scan), and withdrawal of consent. The postoperative MR images needed to be performed within 24 months (±3 months) after arthroscopy.

### 2.2. Patient Characteristics

Of all 42 patients who were arthroscopically treated for a TFCC lesion, 17 patients (40%) with a complete data set and postoperative MRI scan were enrolled in the study. Twenty-five patients had to be excluded because no complete data set was available or the postoperative MRI scan could not be performed (drop-out rate of 60%).

Seven of the patients enrolled were male (41%), 10 were female (59%). The average age of the patients at time of surgery was 35 years (range 16–60 years). Arthroscopic TFCC debridement or arthroscopic-assisted, mini-open TFCC outside-in capsular suture was performed with PDS 4.0 thread. Apart from that, a synovectomy was performed in all patients.

### 2.3. Demographic Target Parameters

Age, gender, pain level PROM scores, and TFCC classification according to the Palmer classification were documented, as was the time interval between surgical treatment and follow-up. Moreover, patients underwent a clinical examination and a MRI scan.

### 2.4. Surgery

All surgical procedures were performed by two senior surgeons. Patients were operated on either under general anesthesia or in plexus anesthesia.

A wrist arthroscopy was performed and existing injuries were addressed. For this purpose, the affected arm was abducted and flexed at 90° in the elbow joint and hung under 2 kg extension using an arthroscopy plate. For arthroscopy, a 2.7 mm, 30° angle optic (Storz KARL STORZ SE & Co. KG Dr.-Karl-Storz-Straße 34, 78532 Tuttlingen, Germany) was used. As arthroscopic portals, the dorsal standard portals were used in the sense of the dorsal 3/4 portal and the 6R portal [10]. Arthroscopic TFCC debridement or arthroscopic-assisted, mini-open TFCC outside-in capsular suture was performed. TFCC lesions were either debrided or sutured depending on the type of rupture. An inside-out suture was performed in case of an ulnar avulsion or tear. For central lesions, a debridement was performed. Apart from that, a synovectomy was performed in all patients.

Classification according to Palmer:

The Palmer classification was used to classify the damage in the TFCC area.

A distinction is made here between traumatic and degenerative damage, each of which is further subdivided.

The traumatic lesions distinguish between a central perforation of the TFCC (type A), an ulnar avulsion (with or without fracture of the distal ulna), which may involve the proximal or distal or both laminae (type B), a distal avulsion of the TFCC involving the ulnotriquetral ligament and the ulnolunar ligament (type C). Type D is defined as a radial avulsion of the TFCC.

The degenerative TFCC lesions are divided into the following types: type A lesions, which describe TFCC wear with thinning but without perforation; type B, which are degenerative changes of the TFCC (type A) with corresponding ulnar, lunar, or triquetral chondromalacia; type C, which refers to degenerative changes with perforation of the TFCC; type D, which is further divided into TFCC perforation with or without chondromalacia and perforation of the lunotriquetral band (with or without characteristics of types A, B, or C); and type E, which describes any or all of the above features with ulnocarpal arthritis

### 2.5. Magnet Resonance Imaging

MR imaging was performed using a 3 Tesla MR scanner (Ingenia, Philips Healthcare, Best, The Netherlands) with a dedicated eight-channel wrist coil (Medical Advances). The following sequences were acquired: triplanar intermediate (IM)-weighted MR sequences with fat suppression, a transversal IM-weighted spectral presaturation with inversion recovery (SPIR) sequence (echo time (TE), 50.0 ms; repetition time (TR), 2419 ms; flip angle, 90°; slice thickness, 2.0 mm; standard field of view (FOV) 80 × 60 × 60 mm^3^), a sagittal IM-weighted SPIR sequence (TE, 50.0 ms; TR, 2567 ms; flip angle, 90°; slice thickness, 2.0 mm; standard field of view (FOV) 80 × 60 × 80 mm^3^), a coronal IM-weighted SPIR sequence (TE, 50.0 ms; TR, 2500 ms; flip angle, 90°; slice thickness, 2.0 mm; standard field of view (FOV) 100 × 40 × 40 mm^3^), and a coronal T1-weighted sequence (TE, 20.0 ms; TR, 614 ms; flip angle, 90°; slice thickness, 2.0 mm; standard field of view (FOV) 100 × 40 × 30 mm^3^). Further details are listed in Table 1.

### 2.6. Patient-Reported Outcome Measures (PROM)

The Munich Wrist Questionnaire (MWQ) was used to investigate wrist function. The MWQ is a validated self-assessment score (this study was performed using the patient-reported outcome measurement (PROM)). PROM was used to measure our main outcome variable, the function. We used the Munich Wrist Questionnaire (MWQ), a validated self-assessment questionnaire that was published in 2016 by M. Beirer et al. [11]. The MWQ addresses questions concerning pain, mobility, and everyday function. Each item is rated with a set score. The maximum achievable score is 250 (100%) and corresponds to full wrist function.

Returned MWQs were evaluated and converted into a percent value. We sent PROM to every arthroscopically treated patient with a TFCC lesion with a request to participate in our study. Received MWQs were checked for missing data or other exclusion criteria. The remaining patients were enrolled in our study and signed up for a MRI scan.

### 2.7. Evaluation of Pain

We divided the study population into two groups depending on the pain they stated in the MWQ. Patients with no pain at all received the maximum score of 50 points. The cut off was set at 40 points.

## 3. Results

### 3.1. Follow-Up

MR imaging mean follow-up was 22 months (range 11–37 months). The average clinical follow-up time of the patients with pain was 27.3 months (range 11–37 months) and 19.5 months (range 14–25 months) for patients without pain.

### 3.2. Classification According to Palmer

All patients had a traumatic TFCC lesion. 1 A is a central slit, 1 B is an ulnar avulsion of the TFCC, and 1 D is a radial avulsion.

Eight (47%) had a TFCC lesion type 1 A, four (23.5%) had type 1 B, and four (23.5%) had type D according to the Palmer classification. Two patients (12%) had massive scarring and a plica at the TFCC without a lesion.

As one patient had both a radial avulsion (1D) and a central slit (1 A), there are 18 pathological findings listed for the 17 patients.

The TFCC lesions type 1 B—ulnar avulsions—were refixed with a mini-open, outside-in capsular suture.

### 3.3. Pain

We divided the study population into two groups depending on the pain they stated in the MWQ. Patients with no pain at all received the maximum score of 50 points. The cut-off was set at 40. Seven patients achieved a pain score ≤40 points, and ten patients had a score >40 points. Two of these patients even presented with severe pain (≤25 points) (Table 2).

### 3.4. MRI Scan

**Figure 1:**
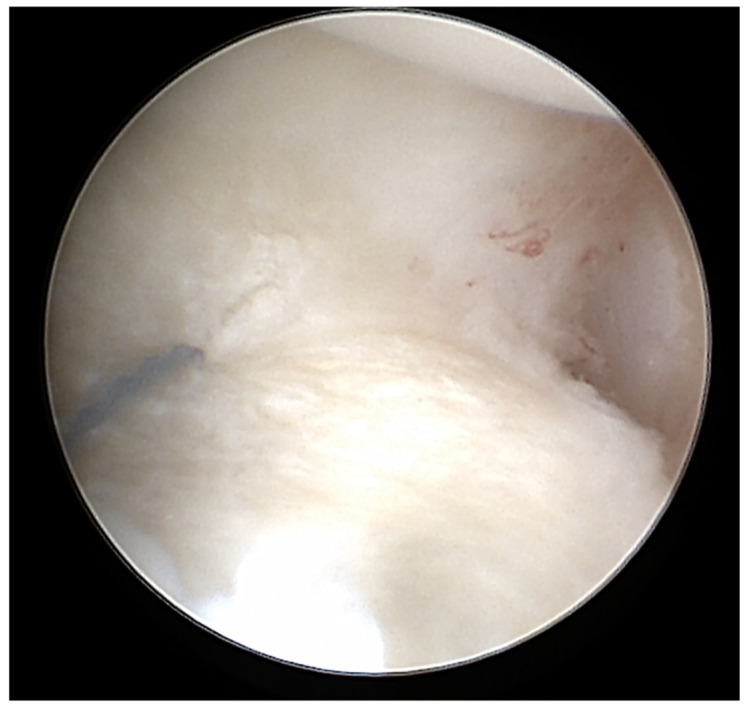
Example for a intraoperative TFCC lesion.

**Figure 2:**
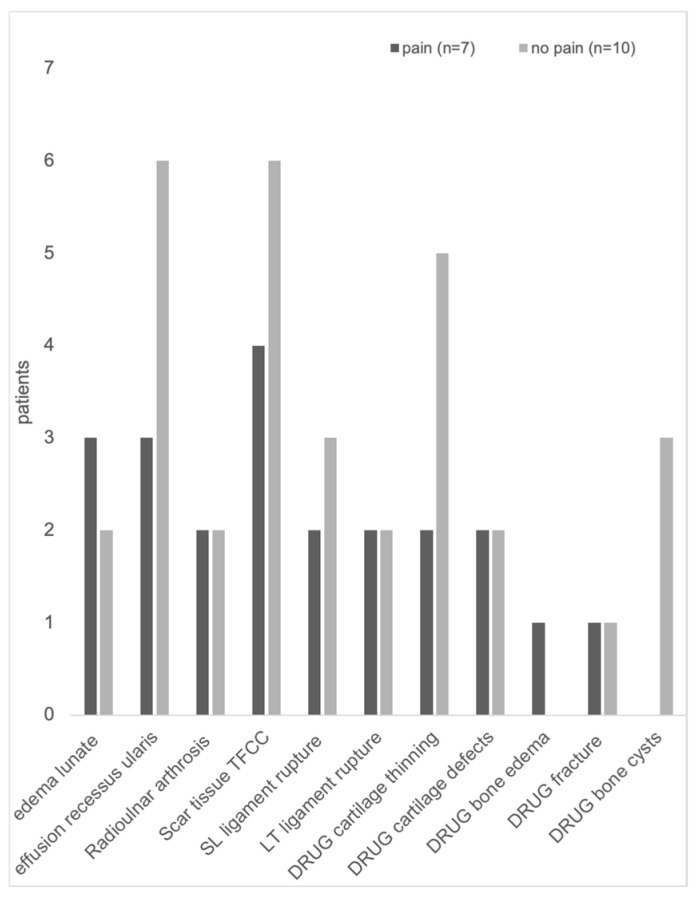
Pathologies detected with MR imaging from patients with pain vs. patients without pain at the wrist after arthroscopy and TFCC surgery.

**Figure 3:**
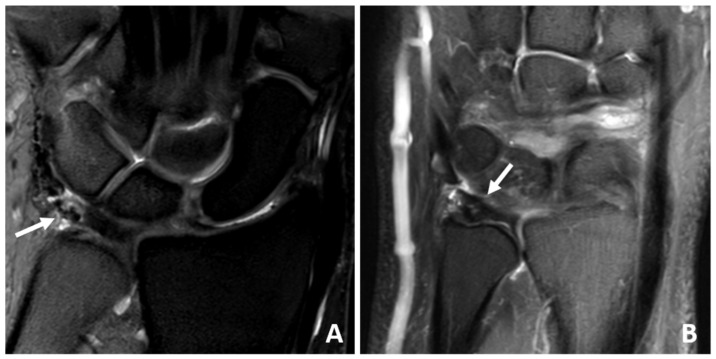
(**A**,**B**) Coronal IM-weighted SPIR sequences of a 53-year-old patient with severe degenerative changes of the TFCC. Note the signal inhomogeneities (arrows) of the TFCC, which are due to scarring and fluid deposition.

**Figure 4:**
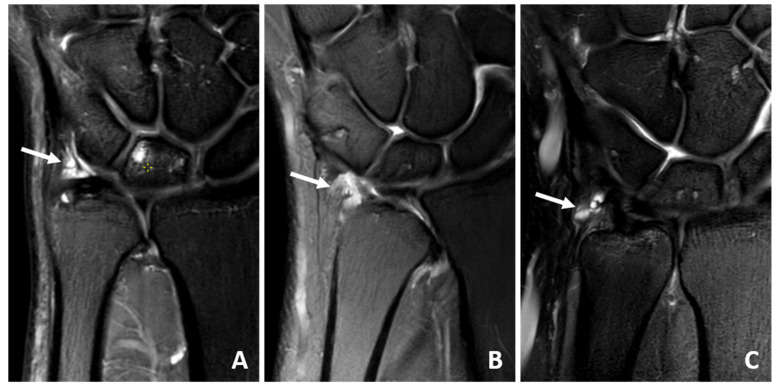
(**A**–**C**) Coronal IM-weighted SPIR sequences of 49 to 61-year-old patients with fluid (arrows) in the ulnar recess after arthroscopy.

Ten patients (59%) had scar tissue at the TFCC (Figure 1, Figure 2 and Figure 3) and nine (53%) had an effusion in the recessus ulnaris (Figure 2 and Figure 4). Both are not necessarily aligned with pain, as six patients without pain and four with pain had scar tissue at the TFCC and six patients without pain and three with pain had an effusion in the recessus ulnaris (Table 3, Figure 4).

**Figure 5:**
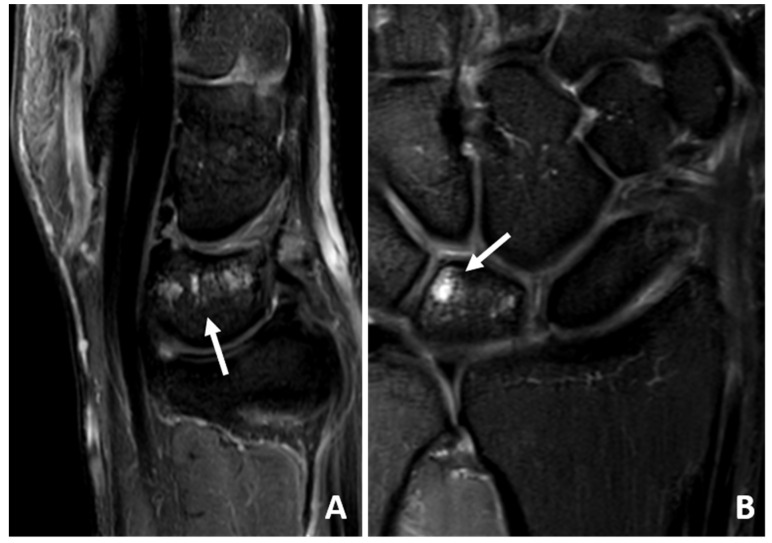
(**A**) Sagittal IM-weighted SPIR sequence and (**B**) coronal IM-weighted SPIR sequence of a 56-year-old patient with bone marrow edema of the lunar bone (arrows).

Bone edema could be found in the lunate of five patients (29%) (three with, two without pain) and in the DRUJ of one patient (6%) with pain (Table 3, Figure 2 and Figure 5).

**Figure 6:**
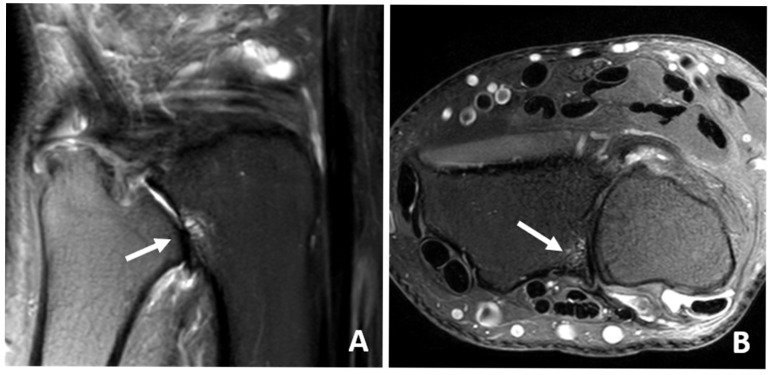
(**A**) Coronal IM-weighted SPIR sequence and (**B**) transversal IM-weighted SPIR sequence of a 62-year-old patient with severe radioulnar arthrosis. Note the typically degenerative findings like joint space narrowing and thinning of the cartilage (arrow in (**A**)) as well as subchondral cyst formation (arrow in (**B**)).

Typical degenerative changes like radioulnar arthrosis and DRUJ cartilage thinning, defects, or cysts were not necessarily associated with pain (Figure 6).

### 3.5. PROM MWQ

**Figure 7:**
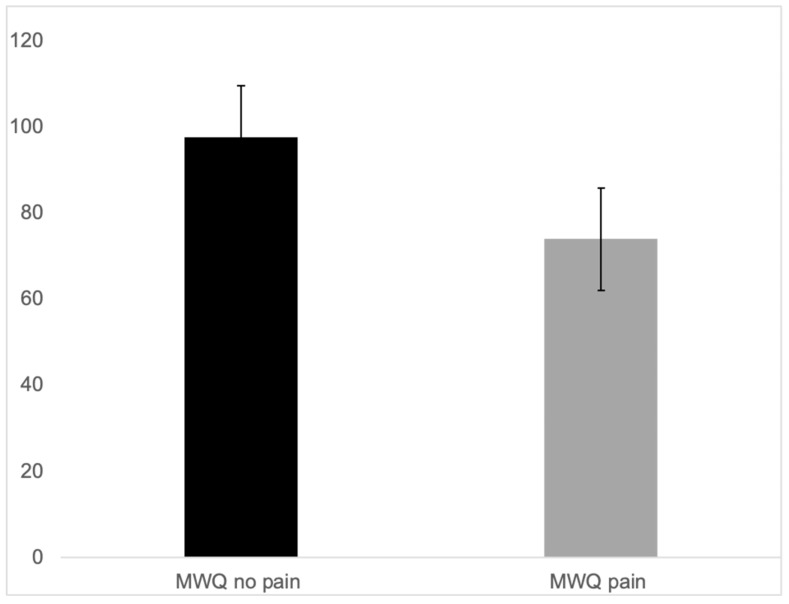
MWQ of the patients with pain (73% average ± 13.9) and MWQ of the pain-free patients (98% average ± 2.1).

The average postoperative function measured with the MWQ was 88% ± 14.8% (average ± SD). Pain impaired function significantly, as the MWQ of the patients with pain was 73% on average (±13.9), whereas the MWQ of the pain-free patients was 98% on average (±2.1) (Figure 7).

## 4. Discussion

TFCC lesions are a common source of ulna-sided wrist pain. The overall results after arthroscopic treatment are good with a high rate of satisfied patients who can fully recover and gain back a painless, full range of motion as well as normal weight bearing of the wrist [12,13,14].

However, some patients returned after arthroscopic surgery of the TFCC because of persisting pain in the wrist. MR imaging, which is performed if pain and loss of function persists and if no improvement of the clinical symptoms can be achieved with conservative treatment options such as physiotherapy, often reveals several potential pathologies. It is still unclear whether these MR imaging findings are of clinical relevance and whether they are a potential reason for the pain or if they are normal postoperative changes.

This study is the first to deal with these postoperative changes in MR imaging and to determine their clinical relevance.

Scarring at the TFCC could be found in 59% of cases, as expected since all patients had surgery at the TFCC. However, imaging could not further specify whether the scar tissue was firm enough to maintain stability or even if it was too tight. It seemed that scar tissue itself at the TFCC alone had no impact on function and pain, as it could be found in four patients with pain and six patients without pain.

However, an edema in the lunate and the DRUJ seemed to impair function and pain. Degenerative changes like radioulnar arthrosis, cartilage thinning at the DRUJ, or a bone cyst in the DRUJ were not associated with pain. This is in contrast to the outcomes of the study from Moloney et al., in which it could be seen that patients with radiocarpal osteoarthritis and no additional surgery after the initial TFCC repair showed significantly worse PROM scores. However, the follow-up examination in this study was performed after 20 years [8], which is a much longer time period compared to the follow-up time of 37 months in this study.

Van der Post et al. analyzed the morphology, homogeneity, and signal intensity of TFCC and TFCC-related MR imaging features in 23 pain-free adolescents with a 3 T MR imaging scanner. They observed several findings in TFCC and TFCC-related features of asymptomatic adolescents in the MRI scan, which could be a normal variation or an asymptomatic anomaly. The rather low inter-observer agreement that was recorded in this previous study needs to be pointed out. It was stated that this should be taken into consideration when interpreting clinical MR images [15]. Therefore, the clinical examination remains the most important feature.

Moreover, regarding the bone edema in the lunate, an ulna impaction syndrome has to be ruled out as this can cause persisting pain. DRUJ instability might be a reason for persisting pain as well and should be checked clinically.

Overall, the results after TFCC surgery are very good, with a MWQ of 88% ± 14.8%. Only two patients had a MWQ lower than 60%. These two patients also stated massive pain (Table 1). The overall good results come along with existing studies, which could show that TFCC repair achieves a good clinical outcome with low complication rates [13].

Other studies often use the DASH score, which is a frequently used questionnaire for the upper extremity. However, the DASH score is rather unspecific for the hand and wrist, which is the reason why we decided to use the MWQ [11]. Moreover, the MWQ includes objective and subjective parameters, thus better reflecting the patients’ situation.

The cut off at 40 points for the pain (maximum score 50 points suggesting no pain) was chosen because patients who still have pain using their hand during daily routines are especially of interest. Severe pain (<25 points) was stated by two patients. While patient number one had several pathological findings, including radioulnar arthrosis, edema in the lunate, an effusion at the recessus ularis, scar tissue TFCC, and a fracture in the bone of the DRUJ (Table 1), in the MRI scan of the second patient, no pathologies could be found.

There are a few studies with long- and mid-term results after arthroscopic treatment of TFCC lesions. The median follow-up time ranges from 11 months to 20 years [1,2,4,5,8]. The mean follow-up time in this present study was 22 months (range 11–37 months). It is interesting that in the patient group with pain, the follow-up was longer (27 months on average) compared to the pain-free group (19.5 months).

The limitations of this study are the small patient number. The results of this study need to be confirmed in a larger study population.

There are several different surgical methods for TFCC repair (open, all arthroscopic, arthroscopic-assisted, mini-open, no-suture, outside-in, etc.). All patients enrolled in this present study have been treated with an arthroscopic-assisted, mini-open TFCC repair outside-in. Accordingly, the statement concerning the outcome cannot be easily transferred to all other TFCC repairs, as the literature could show that there was a huge inhomogeneity due to different surgical techniques [5,13].

## 5. Conclusions

In conclusion, this present study is the first to assess the outcome after arthroscopic-assisted TFCC surgery both regarding the pain and function of the patient and the associated abnormal findings in the MRI scan.

Some postoperative MRI findings like bone edema in the lunate or in the DRUJ might be more likely to cause pain or impaired function than others (scar tissue at the TFCC or effusion in the recessus ulnaris, for example). Degenerative changes may cause long-term pain due to arthrosis development, as studies with a long-term clinical follow-up showed [7,8]. However, as this study and others before [15] show that not all pathological findings are associated with clinical symptoms, the clinical examination remains a very important indicator regarding the decision as to whether a surgical revision should be performed.

## Figures and Tables

**Table 1:** Sequence parameters.

Sequence	Axial IM/SPIR	Sagittal IM/SPIR	Coronal IM/SPIR	Coronal T1
Echo time (ms)	50	50	50	20
Repetition time (ms)	2419	2567	2500	614
Flip angle	90	90	90	90
Field of view (mm^3^)	80 × 60 × 60	80 × 60 × 80	100 × 40 × 40	100 × 40 × 40
Slice Thickness (mm)	2	2	2	2
Slice number	28	20	27	18

**Table 2:** Patient characteristics, imaging, and clinical findings of patients with severe pain.

	Gender	Time After Surgery (Months)	Age	Pathological Findings on MR Images	Pain Score	MWQ (%)
Patient 1	Female	36	45	Edema lunate Effusion recessus ularis Radioulnar arthrosis Scar tissue TFCC DRUJ fracture in the bone	25	59
Patient 2	Female	19	24	\	16	58

**Table 3:** Results of imaging: TFCC = triangular fibrocartilage complex, DRUJ = distal radioulnar joint, SL Ligament = scapholunate ligament, LT ligament = lunotriquetral ligament).

	Patients with Pain n = 7 (41%) [n]	Patients Without Pain n = 10 (59%) [n]	Percentage of the Study Population n = 17 [n]
Edema lunate	3 (43%)	2 (20%)	5 (29%)
Effusion recessus ularis	3 (43%)	6 (60%)	9 (53%)
Radioulnar arthrosis	2 (29%)	2 (20%)	4 (24%)
Scar tissue TFCC	4 (57%)	6 (60%)	10 (59%)
SL ligament rupture	2 (29%)	3 (30%)	5 (29%)
LT ligament rupture	2 (29%)	2 (20%)	4 (24%)
DRUJ cartilage thinning	2 (29%)	5 (50%)	7 (41%)
DRUJ cartilage defects	2 (29%)	2 (20%)	4 (24%)
DRUJ bone edema	1 (14%)	0	1 (6%)
DRUJ fracture	1 (14%)	1 (10%)	2 (12%)
DRUJ bone cysts	0	3 (30%)	3 (18%)

## Data Availability

The data and materials are available from the corresponding author upon a reasonable request.

## Abbreviations

The following abbreviations are used in this manuscript:

TFCC	Triangular fibrocartilage complex
PROM	Patient-reported outcome measurement
MRI	Magnet resonance imaging
MWQ	Munich Wrist Questionnaire
DRUJ	distal radioulnar joint
